# The Spectrum of Cutaneous Manifestations in Dermatomyositis: A Comprehensive Review

**DOI:** 10.3390/jcm15082874

**Published:** 2026-04-10

**Authors:** Magdalena Kutwin, Paulina Karp, Marcelina Kądziela, Alicja Siennicka, Agnieszka Żebrowska

**Affiliations:** Department of Dermatology and Venereology, Medical University of Lodz, Haller sq. 1, 90-647 Lodz, Poland

**Keywords:** dermatomyositis, idiopathic inflammatory myopathies, juvenile dermatomyositis, drug-induced dermatomyositis, hypomyopatic dermatomyositis, amyopathic dermatomyositis

## Abstract

Dermatomyositis (DM) is a rare, autoimmune inflammatory myopathy characterized by a heterogeneous clinical course and complex etiopathogenesis. Although classically defined by the coexistence of muscle inflammation and distinctive skin lesions, DM frequently presents as a systemic disease, and, in some patients, cutaneous manifestations may precede muscle involvement or represent the sole clinical feature. The spectrum of skin lesions in DM is broad and includes pathognomonic, characteristic, rare, or atypical manifestations, ranging from classic Gottron’s sign and heliotrope rash to uncommon subtypes such as vesiculobullous dermatomyositis, Wong-type dermatomyositis, or flagellate dermatitis. Particular cutaneous phenotypes often correlate with distinct clinical subtypes, autoantibody profiles, systemic involvement, and prognosis. The diversity of dermatological presentations and their resemblance to other dermatoses may delay accurate diagnosis, especially in amyopathic and hypomyopathic forms of the disease. The aim of this review is to comprehensively discuss the wide spectrum of cutaneous manifestations of dermatomyositis, emphasize less recognized and rare dermatological clinical presentations, and highlight their diagnostic and prognostic significance. However, histopathological examination may support the diagnostic process in challenging cases. Early identification of characteristic skin lesions remains crucial for prompt diagnosis, appropriate screening for systemic complications and malignancy, and optimal management. Close interdisciplinary cooperation among dermatologists, rheumatologists, and other specialists is essential to ensure accurate diagnosis and improve outcomes in patients with dermatomyositis.

## 1. Introduction

Idiopathic inflammatory myopathies (IIMs) are a heterogeneous group of rare, chronic autoimmune diseases, including dermatomyositis (DM), polymyositis (PM), inclusion body myositis (IBM), and other less common subgroups of myositis.

DM, according to the nomenclature, is characterized by inflammation of the skeletal muscles and skin involvement [1,2,3]. The most common symptom of DM is gradually progressive, painful, symmetrical proximal muscle weakness. However, skin lesions may precede muscle weakness by months and may even be the only symptom of the disease [4]. However, DM typically involves other organs and systems, demonstrating significant heterogeneity in clinical presentation and biochemical features, as well as a variable response to treatment.

The literature distinguishes several types of DM, starting with the most common, so-called classical form of DM, through to the childhood form of DM (juvenile dermatomyositis—JDM), the paraneoplastic form of DM (paraneoplastic dermatomyositis—PDM), drug-induced DM (drug-induced dermatomyositis—DIDM), DM without muscle involvement (dermatomyositis sine myositis, clinically amyopathic dermatomyositis—CADM), and ending with the oligosymptomatic form in relation to muscle involvement (hypomyopatic dermatomyositis—HDM) [4].

The diagnostic criteria for DM and PM, proposed by Peter and Bohan in 1975, formed the basis for the classification of idiopathic inflammatory myopathies for many decades [3,5]. In 2017, experts from the European League Against Rheumatism (EULAR) and the American College of Rheumatology (ACR) proposed new classification criteria for idiopathic inflammatory myopathies (IIM), which also include dermatomyositis (Table 1) [6].

These criteria were developed to standardize and improve diagnostic accuracy while reducing the number of false positives. The classification system is based on a scoring system of clinical symptoms, laboratory test results, and histopathological data. A score exceeding 6.7 points in patients with a muscle biopsy, or 5.5 points in the absence of a muscle biopsy, allows for classification as probable IIM. Values exceeding 8.7 and 7.5 points, respectively, constitute a reliable diagnosis of IIM [6]. In the case of dermatomyositis, in addition to meeting the classification criteria for IIM, the simultaneous presence of at least one characteristic skin symptom listed in Table 1 is required (bold text). In the presence of skin lesions typical of DM, histopathological confirmation of muscle involvement is not essential for diagnosis; it is merely optional.

In addition to the tests included in the EULAR/ACR classification system, the diagnosis of DM may be enhanced with additional supportive tests. These include nonspecific screening tests (e.g., erythrocyte sedimentation rate or C-reactive protein level), more targeted immunological tests (e.g., IIM-specific autoantibodies, antibodies against Ro/SSA and La/SSB, antibodies against PM-Scl), imaging studies (e.g., electromyography, magnetic resonance imaging), and tests for malignancy [4,7].

IIMs are rare diseases, with DM being the most common form, except in the elderly population, where inclusion body myopathy predominates [8]. In childhood, the most frequently diagnosed IIM is juvenile dermatomyositis (JDM), accounting for approximately 85% of all cases [9]. DM can occur at any age, with a two-peak incidence between ages 5–15 and 40–60 [10,11,12]. The disease is more common in women, with a female predominance ratio of approximately 2:1 [8]. The exact pathogenesis of DM remains unknown, but it is considered an autoimmune disease in which a key role is played by a dysfunctional immune response, leading to vascular damage in skeletal muscle and skin. Autoantibodies in idiopathic inflammatory myopathies can be classified into two groups: myositis-specific autoantibodies (MSAs), detected in approximately 50% of patients, and myositis-associated autoantibodies (MAAs), which occur in approximately 20% of patients with IIM [13].

MSAs are highly specific for these conditions and often correlate with particular clinical phenotypes of DM, whereas MAAs are less typical and can also occur in other systemic connective tissue diseases. IIM-specific autoantibodies (MSAs) include anti-tRNA synthetase antibodies (ASAb), anti-MDA5 (anti-melanoma-associated protein 5, also known as anti-CADM140), anti-Mi-2 (anti-nuclear helicase), anti-SRP (anti-signal recognition particle), anti-TIF1 (anti-transcriptional intermediary factor 1, also known as anti-p155/p140), anti-NXP2 (antinuclear matrix protein 2), and anti-SAE (anti-SUMO-1 activating enzyme) [13].

Regarding antisynthetase antibodies, autoantibodies directed against eight of the twenty amino acids have been identified. These include anti-Jo-1 (anti-histidyl-tRNA), which is the most common, and anti-PL-7 (anti-threonyl-tRNA), anti-PL-12 (anti-alanyl-tRNA), anti-OJ (anti-isoleucyl-tRNA), anti-EJ (anti-glycyl-tRNA), anti-KS (anti-asparaginyl-tRNA), anti-Zo (anti-phenylalanyl-tRNA), and anti-Ha (anti-tyrosyl-tRNA, also known as anti-YRS) [14,15,16,17,18,19,20,21]. Interactions between genetic predisposition, environmental factors, and immunological effector mechanisms also play a role in the pathogenesis, which determines the heterogeneous clinical picture of DM [22,23].

This review aims to discuss the spectrum of cutaneous manifestations of DM, ranging from mild to severe, and to draw the attention of physicians of various specialties to the less common and less recognizable clinical presentations of DM and the fact that skin lesions may be the only symptom of the disease and persist long after treatment has been completed.

## 2. Materials and Methods

**Scheme 1:**
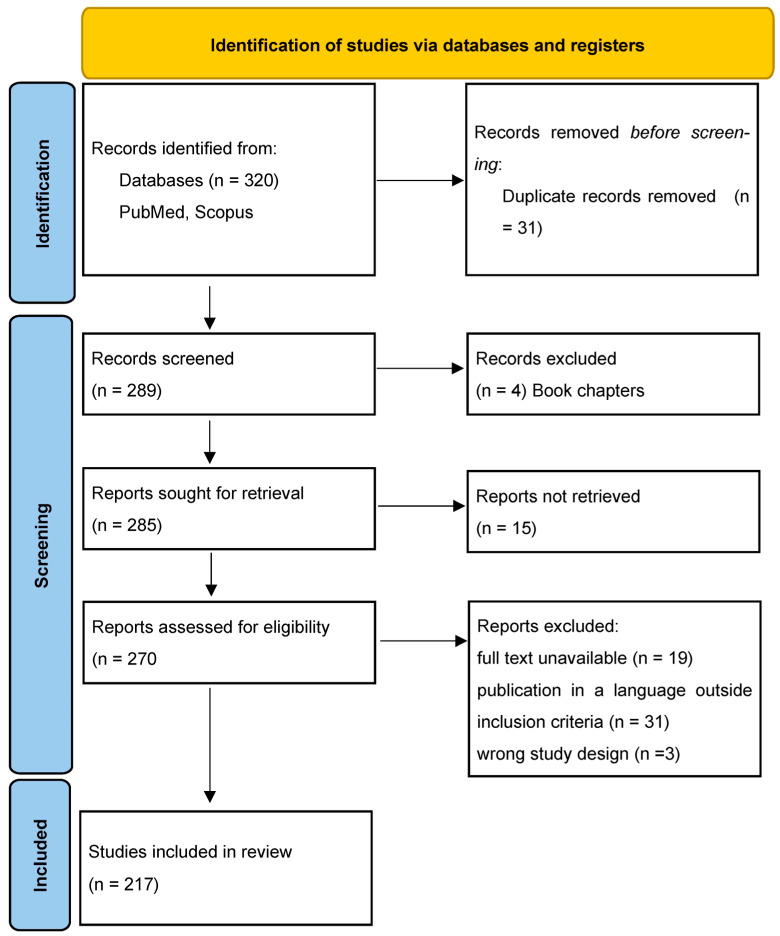
Description of the methodology for selecting sources for this review article.

In November 2025, a research study on the spectrum of cutaneous manifestations was conducted by M.K., P.K., M.K., A.S., and A.Ż. All reviewers independently executed the search strategy across the selected electronic databases and assessed each study. Titles and abstracts of retrieved records were screened independently by all reviewers, and full-text articles were subsequently assessed for eligibility. Any disagreements between reviewers at any stage of the screening or selection process were resolved through discussion and consensus. The literature search was performed in the PubMed and Scopus databases using the following English-language keywords: “dermatomyositis”, “cutaneous manifestations”, “autoimmune inflammatory myopathy”, “systemic inflammatory disease”, “amyopathic dermatomyositis”, “hypomyopathic dermatomyositis”, “myositis-specific autoantibodies”, “idiopathic inflammatory myopathies”, “Gottron’s papules”, “heliotrope rash”, “shawl sign”, “V-sign”, “mechanic’s hands”, “vesiculobullous dermatomyositis”, “Wong-type dermatomyositis”, “flagellate dermatitis”, “paraneoplastic syndromes”, “malignancy-associated dermatomyositis”, “diagnostic challenges”, “histopathology”, and “prognostic markers”. All retrieved records were manually assessed, and only studies meeting this criterion were included in the final analysis, ensuring comprehensive identification of relevant literature while minimizing the risk of omission. Articles written in English were included, and in total, 320 potentially eligible articles were detected. Additional sources were retrieved by examining the reference lists of selected articles. After eliminating duplicates, non-relevant entries based on titles and abstracts, and excluding book chapters, a final total of 217 articles was included in this review Scheme 1.

## 3. Types of Dermatomyositis

### 3.1. Classical Dermatomyositis

#### 3.1.1. Dermatological Findings

The presence of typical skin findings is essential for establishing a diagnosis of DM [5]. In over half of affected patients, dermatological signs appear months or even years before the onset of muscle weakness [24]. Skin involvement in DM can be categorized into pathognomonic, characteristic, and compatible features based on their diagnostic specificity. Furthermore, other dermatological manifestations have been documented, including rare and nonspecific signs [1,25].

#### 3.1.2. Pathognomonic Cutaneous Features

**Figure 1:**
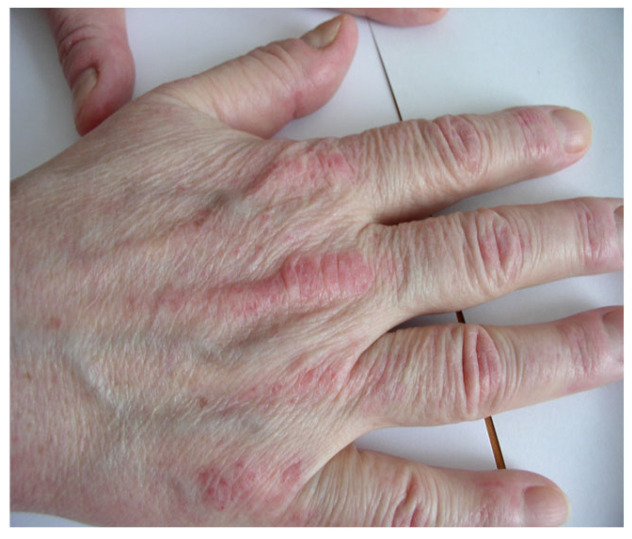
Gottron’s papules and Gottron’s sign.

**Figure 2:**
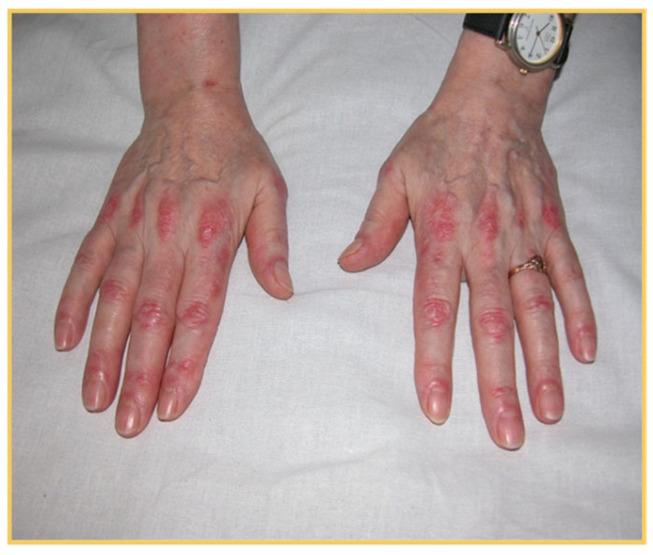
Gottron’s papules and Gottron’s sign.

The pathognomonic skin lesions of classic DM include Gottron’s papules and Gottron’s sign [25]. Gottron’s papules present as violaceous, slightly elevated papular lesions arising on an erythematous base. They are typically located on extensor surfaces overlying bony prominences, particularly the metacarpophalangeal and proximal and distal interphalangeal joints, and may extend to involve the periungual regions [25,26] (Figure 1 and Figure 2). Gottron’s sign refers to flat erythematous macules, often arranged linearly, affecting the dorsal and lateral parts of the hands and fingers. These lesions frequently undergo subsequent scaling and may also be observed on other extensor sites, including the elbows and knees [25].

#### 3.1.3. Characteristic Cutaneous Features

**Figure 3:**
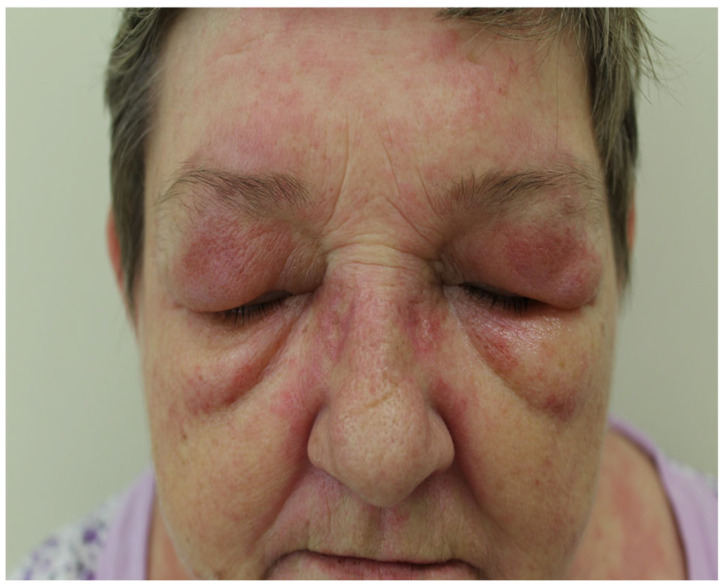
Heliotrope rash with periorbital edema.

**Figure 4:**
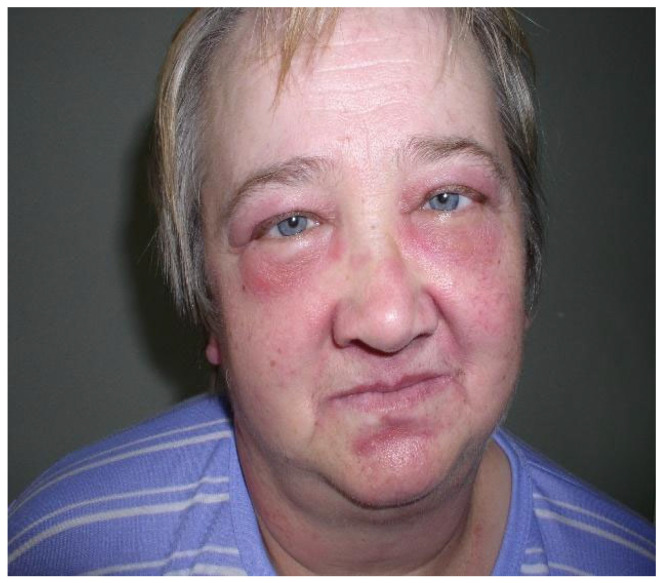
Heliotrope rash with periorbital edema.

Characteristic skin findings include heliotrope rash, shawl sign, V-sign, nailfold abnormalities (Keining’s sign), and scalp dermatitis [27]. Heliotrope rash is characterized by symmetrical violaceous erythema, at times with accompanying oedema, predominantly affecting the upper eyelids and often associated with pruritus. Extension to the cheeks, nasal bridge, and nasolabial folds may occur. In some individuals, the presentation is limited to subtle eyelid discoloration or dilated eyelids vessels. Patients with lupus erythematosus (LE) and scleroderma may also rarely present heliotrope rash [28] (Figure 3 and Figure 4). 

**Figure 5:**
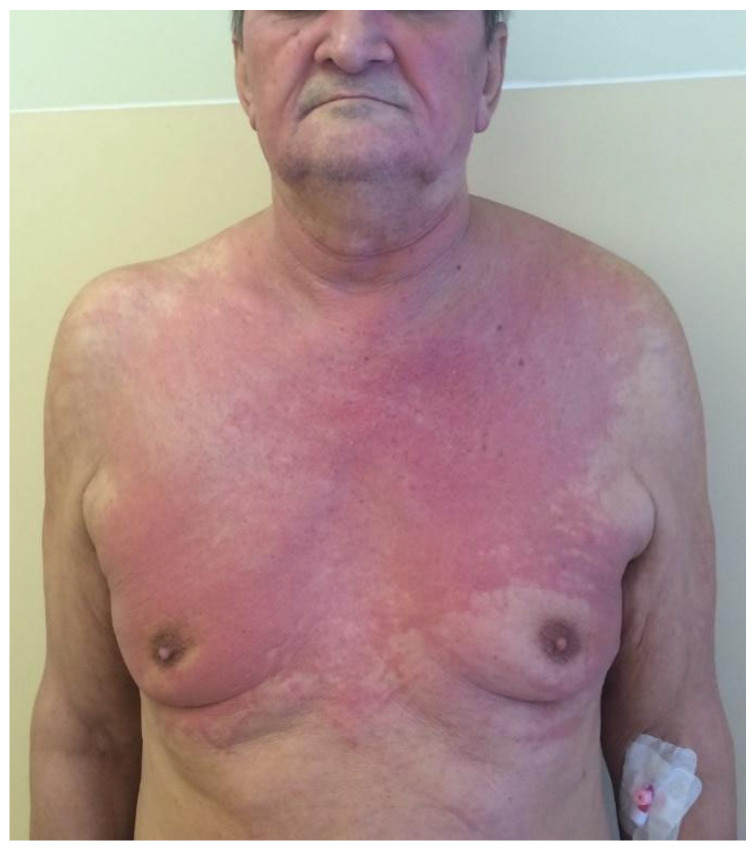
Shawl sign and V-sign.

**Figure 6:**
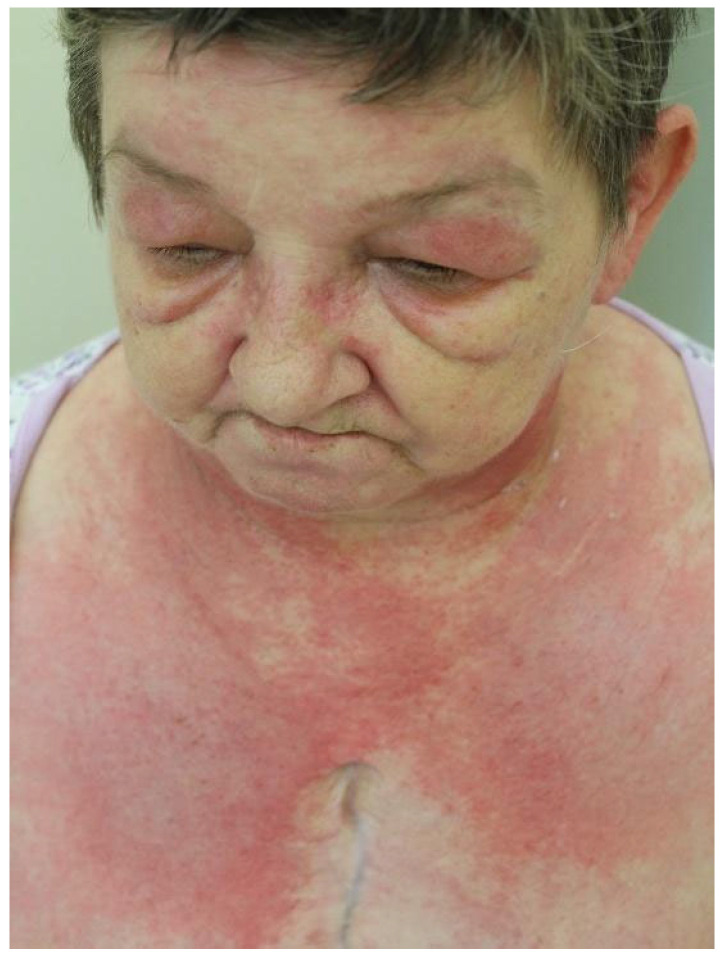
Shawl sign and V-sign.

The shawl sign presents as an erythematous maculopapular eruption over the neck, upper back, and shoulders, whereas the V-sign involves a similar rash distributed over the neck and anterior upper chest in a V-shaped pattern [27,29,30] (Figure 5 and Figure 6).

**Figure 7:**
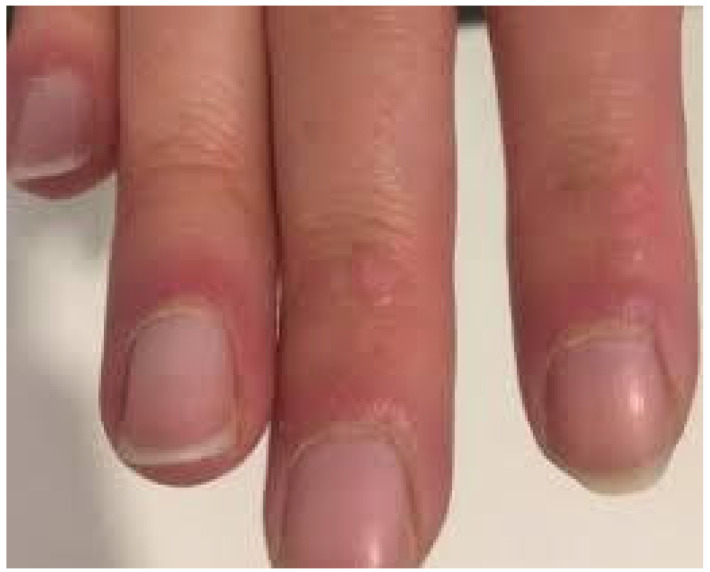
Nailfold abnormalities.

**Figure 8:**
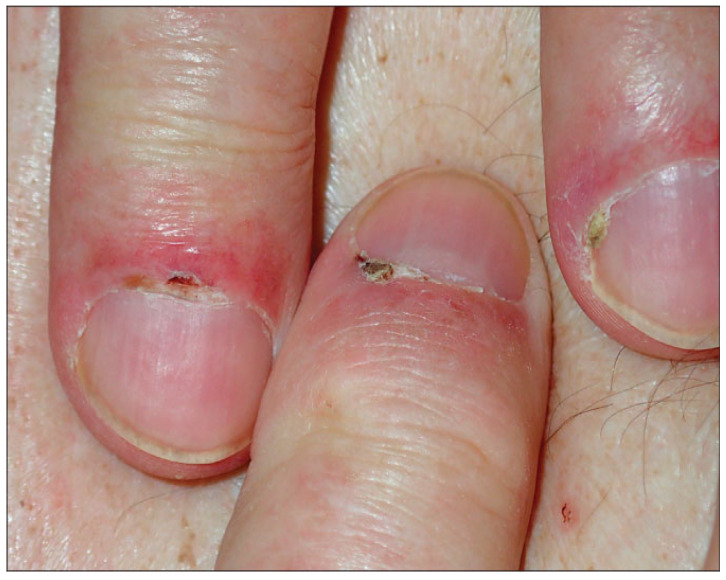
Nailfold abnormalities and erosions.

Dermatomyositis may be associated with distinctive nailfold abnormalities, supporting the potential diagnostic value of nailfold capillaroscopy in affected patients. Furthermore, higher capillaroscopic scores have been shown to correlate with disease activity in patients with DM [31,32]. Capillaroscopic evaluation in DM most commonly reveals an increased vascular diameter, decreased capillary density, scleroderma-spectrum patterns, and microhaemorrhages [31]. Keining’s sign refers to characteristic nailfold abnormalities observed in dermatomyositis, including periungual telangiectasia, cuticular hypertrophy or dystrophy, and punctate hemorrhages, reflecting underlying microvascular involvement [32] (Figure 7 and Figure 8).

**Figure 9:**
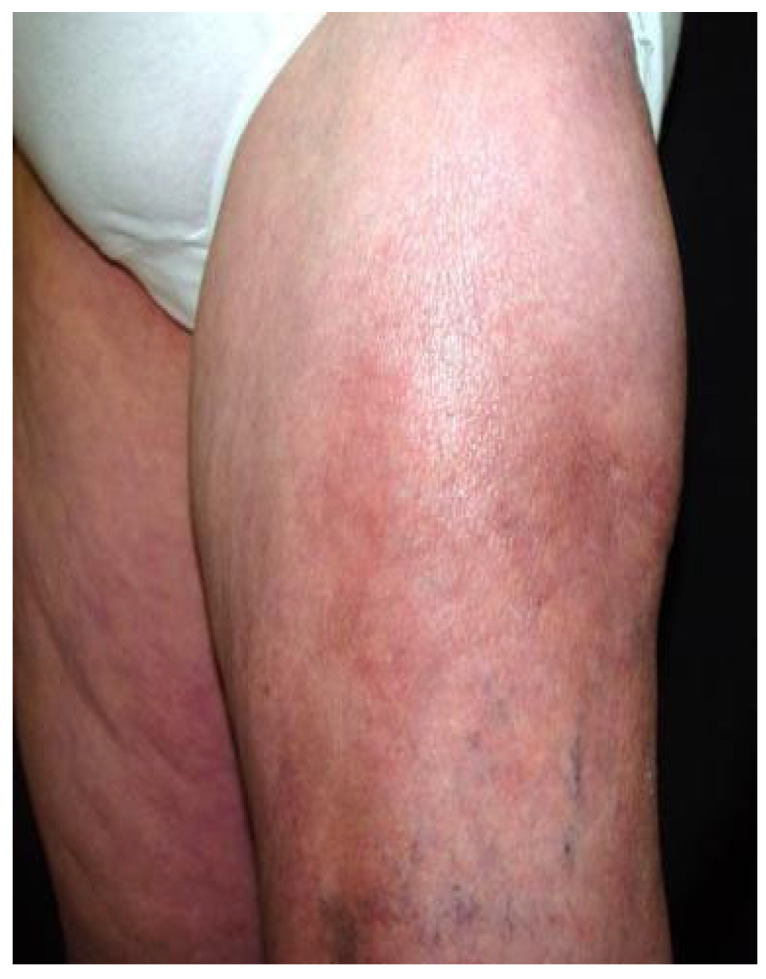
Holster sign.

**Figure 10:**
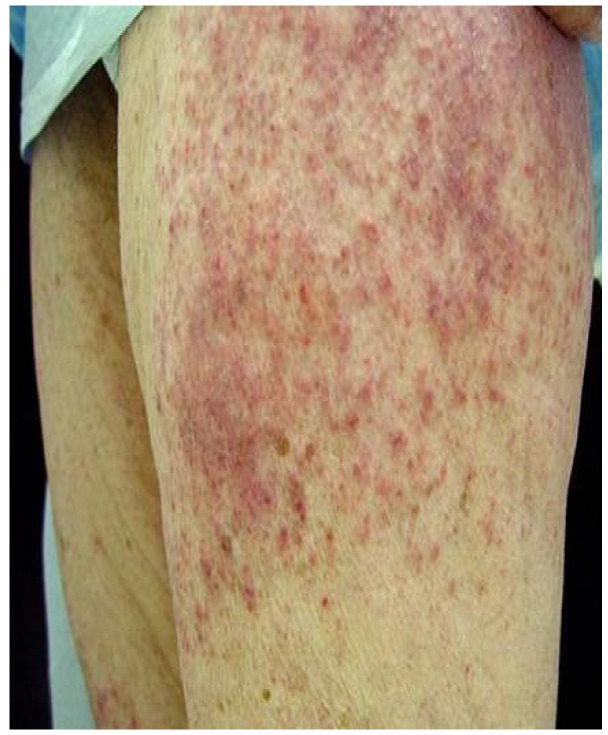
Holster sign.

**Figure 11:**
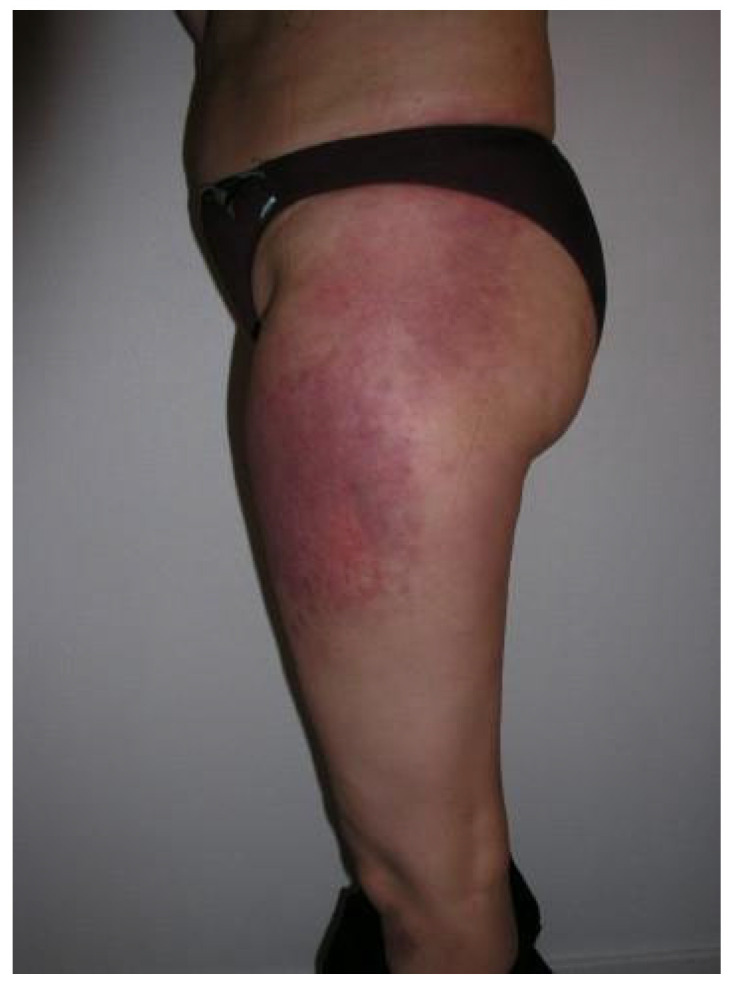
Holster sign.

Compatible 9. represents poikiloderma, localized to the lateral sides of thighs and hips [30] (Figure 9, Figure 10 and Figure 11). Periorbital violaceous oedema may be bilateral and contribute to diffuse facial swelling [30].

#### 3.1.4. Less Common Cutaneous Features

**Figure 12:**
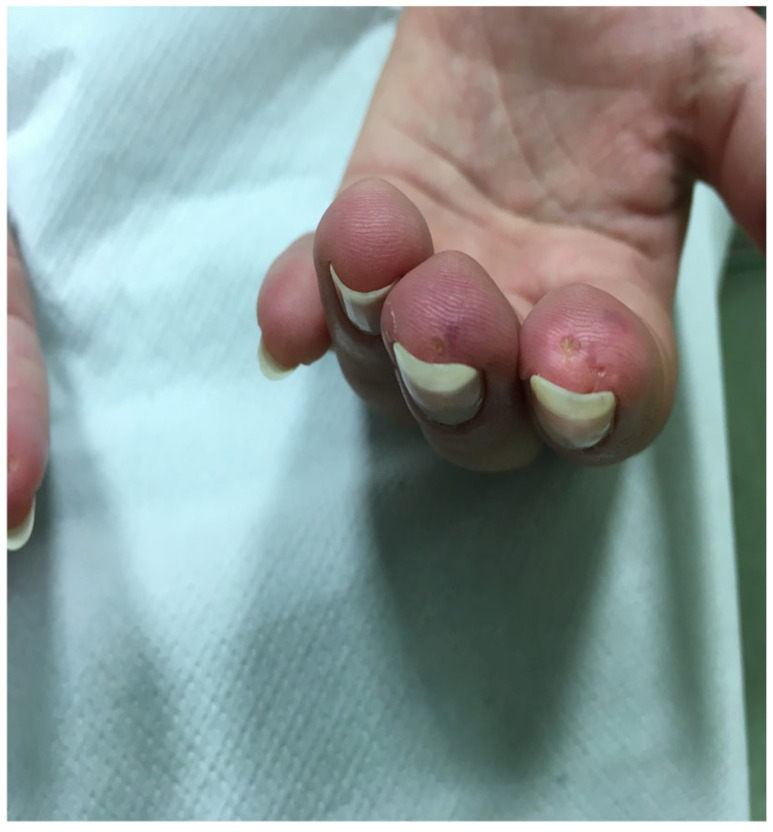
Vasculitis skin changes.

**Figure 13:**
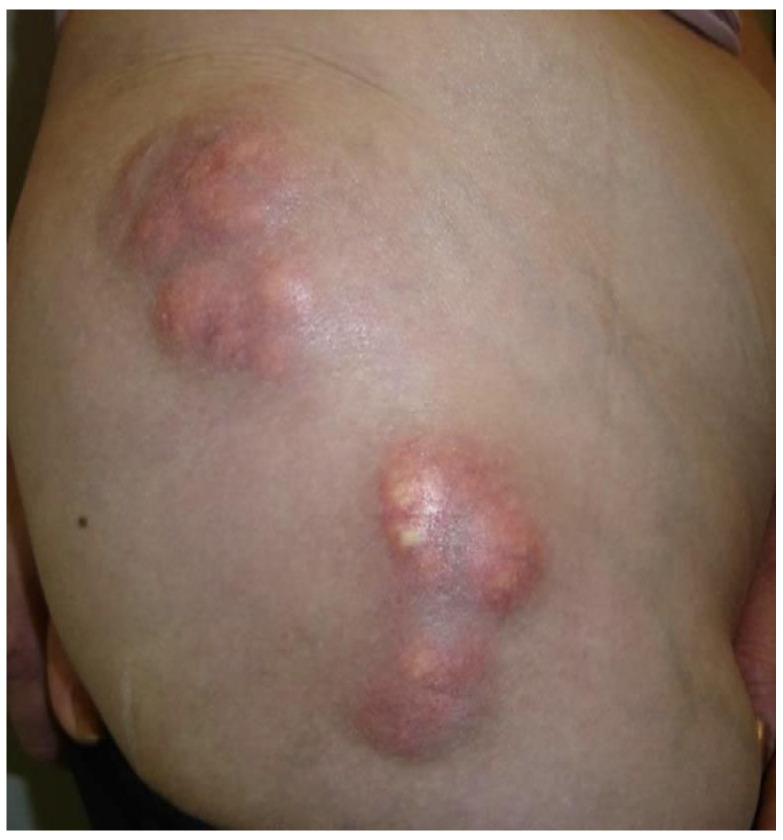
Calcinosis cutis.

Cutaneous vasculitis and calcinosis cutis are less frequent but clinically significant features of DM. Vasculitic involvement may present with vesicles, necrosis, and erosions, and is more commonly reported in juvenile dermatomyositis (JDM) than in adults. Other manifestations include palpable purpura, urticarial eruptions, livedo reticularis, and mucosal or digital ulcerations. In adults, vasculitic skin changes have been associated with underlying malignancy [27]. Calcinosis cutis is characterized by calcium deposition within the skin and subcutaneous tissues, forming firm nodules most often located on the elbows, knees, and buttocks. While it affects up to 70% of patients with JDM, it occurs in approximately 10% of adults with DM [33]. Extrusion of calcium deposits may result in chronic, non-healing ulcers [27]. In adults, calcinosis has been linked to both solid and hematologic malignancies [33] (Figure 12 and Figure 13).

#### 3.1.5. Rare Cutaneous Features

**Figure 14:**
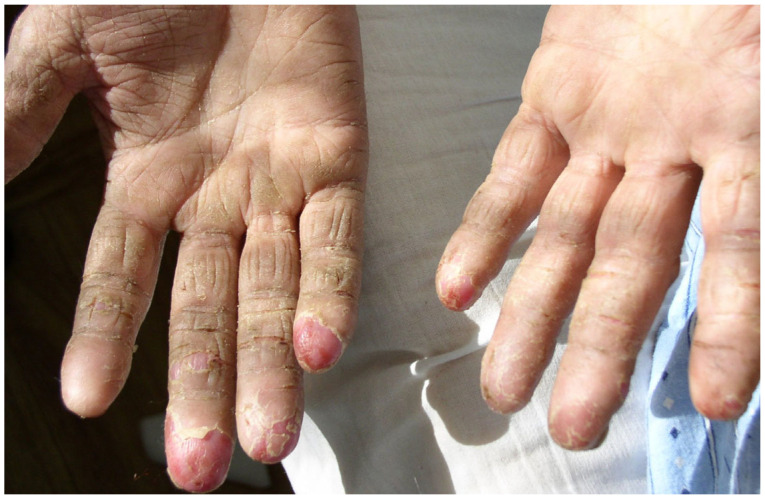
Mechanic’s hand.

DM is notable for its wide spectrum of dermatological presentations. Rare manifestations include mechanic’s hands, flagellate erythema, panniculitis, cutaneous mucinosis, inverse Gottron’s papules, erythroderma, and oral mucosal involvement [27,33]. Mechanic’s hands are characterized by hyperkeratosis, fissuring, and scaling of the lateral aspects of the fingers, and occasionally the palms of the hands (Figure 14). It can resemble contact dermatitis [25,29]. Flagellate erythema consists of linear, erythematous streaks resembling whip marks, typically affecting the back, lateral chest, or upper buttocks [25,30]. Panniculitis is a rare presentation of DM that usually involves the thighs and buttocks, initially presenting with erythema and potentially progressing to subcutaneous calcification and lipodystrophy, particularly in juvenile patients. Histopathological examination typically reveals lobular panniculitis with dense lymphocytic and plasmacellular infiltration [25,34]. Cutaneous mucinosis may manifest as erythematous papules, plaques, or scleromyxedema-like features. Erythroderma, although uncommon, has been reported in association with DM and may signal an underlying neoplastic process [30]. Inverse Gottron’s presents as painful papules or erythema on the palmar surfaces of the hands. This sign is very rare and may be associated with interstitial lung disease [25]. Vescico-bullous skin lesions have been rarely documented in patients with DM. In these cases, the incidence of internal malignancies is much higher than in other cutaneous presentations of DM [25,35]. In the paper by Kubo et al. [36] 19 cases of DM with vesiculobullous lesions were reviewed. All except for one case revealed subepidermal blisters; in one case, intraepidermal blisters were found [37]. Oral involvement includes gingival telangiectasia, mucosal erosions or ulcers, xerostomia, and leukoplakia [30,36,38].

***Antibodies.***

Myositis-specific autoantibodies—despite their clinical relevance, the role of these antibodies in the pathogenesis of DM remains unclear [3,5]. DM-specific antibodies include anti-Mi2, anti-MDA5, anti-NXP2, anti-TIF1, and anti-SAE [23].

#### 3.1.6. Anti-Mi2

Anti-Mi-2 antibodies target a nuclear DNA helicase involved in transcription. Their prevalence among adult patients with DM ranges from approximately 4% to 35% [23]. The presence of anti-Mi-2 antibodies is associated with a classic DM phenotype, characterized by the development of pathognomonic cutaneous manifestations. Additionally, other typical dermatological findings most commonly linked to anti-Mi-2-positive DM include facial dermatosis, heliotrope rash, and the shawl sign, whereas more severe cutaneous complications, such as calcinosis and ulcerative vasculopathy, are infrequently observed in this subset [1,23,25].

#### 3.1.7. Anti-TIF1

Transcription intermediary factor 1 (TIF1) is a tumor suppressor protein. Autoantibodies directed against TIF1 are detected in approximately 18–23% of adult patients with DM [39]. These antibodies have been linked to both solid tumors and hematologic cancers, with reported malignancy rates ranging from 20% to 65% [39,40]. In addition to malignancy risk, anti-TIF1-γ-associated DM is characterized by prominent cutaneous involvement, including marked photosensitivity, heliotrope rash, the V-sign, Gottron’s papules, palmar hyperkeratosis, atrophic hypopigmented patches with teleangiectasia, and ovoid palatal patches [23,25,41,42]. These patients often exhibit more severe muscle weakness and commonly gastrointestinal involvement. However, anti-TIF-1γ was associated with a lower prevalence of Raynaud phenomenon and arthritis in DM patients [23,41,43].

#### 3.1.8. Anti-MDA5

Anti-melanoma differentiation-associated gene 5 antibodies (anti-MDA5) target an RNA-specific helicase that plays a key role in antiviral innate immune responses, including the induction of type I Interferon [44]. In clinically amyopathic dermatomyositis (CADM), these antibodies are present in most adult and pediatric patients, while their prevalence among all individuals with dermatomyositis ranges from approximately 10% to 30% [45,46,47]. The presence of anti-MDA5 antibodies is strongly associated with an increased risk of ILD [44,45]. Cutaneous manifestations are often severe and may include painful ulcerations, particularly involving acral sites, periungual areas, elbows, and knees, as well as palmar papules and digital ischemia [48].

#### 3.1.9. Anti-NXP-2

Nuclear matrix protein 2 (NXP-2) is involved in the regulation of transcription and RNA metabolism [44]. Anti-NXP-2 autoantibodies are detected in a relatively small proportion of adult patients with DM, with reported prevalence varying by ethnicity (2–25%) [41,47]. The presence of anti-NXP-2 antibodies in adults is associated with an increased risk of malignancy [40]. Clinically, anti-NXP-2-associated DM generally presents with edema, calcinosis, and distal ulcerations, which have been more frequently reported in affected patients [23,41].

#### 3.1.10. Anti-SAE

Autoantibodies against the small ubiquitin-like modifier activating enzyme (anti-SAE) are detected in approximately 6–8% of adult patients with DM [13]. Clinically, anti-SAE-positive DM is characterized by prominent and often severe cutaneous involvement at disease onset, accompanied by little or no initial muscle weakness. [49].

#### 3.1.11. Histopathology

Histopathological evaluation of skin biopsies from dermatomyositis lesions may not be distinguishable from skin lesions in systemic lupus erythematosus (SLE). Still, it is important in the process of excluding certain diseases from the differential diagnosis [50,51,52]. The most commonly observed histopathological features in DM include excessive mucin deposition in the dermis, mild to moderate inflammatory infiltrates composed of mononuclear cells, and vacuolization of the basal cell layer [50,51]. Pathognomonic skin lesions in patients with DM do not present with a similarly unique histopathological picture of the specimen [53].

#### 3.1.12. Systemic Manifestations

Despite various skin manifestations of dermatomyositis, the course of the disease is not limited to these symptoms alone. As a result of a generalized inflammatory process, many other systems are also affected, such as the cardiovascular, respiratory, muscular, and digestive systems, which often give the disease a systemic character [54]. It is believed that cardiac involvement in dermatomyositis involves functional or structural abnormalities of the heart that result from the chronic inflammatory process characteristic of this disease. This process can affect various structures of the heart, leading to the development of numerous cardiovascular complications [55]. The most common causes of death in the course of this disease include conduction disorders and arrhythmias, congestive heart failure, valve, pericardial, and coronary artery diseases, and left ventricular dysfunction [56,57]. Pulmonary manifestations of DM are among the most important extra-cutaneous complications that have a significant impact on the course of the disease. The most commonly observed form is ILD, which can lead to progressive fibrosis, respiratory failure, and an increased risk of death [58,59,60]. Although less common than cardiac and pulmonary changes, symptoms affecting the digestive system are also present. The most common changes that accompany dermatomyositis are dysphagia, which is associated with dysfunction of the throat and esophagus, and disturbances in motility of the gastric and small intestine. These disorders can significantly worsen the patient’s condition, leading to malnutrition and other complications [23,61,62,63].

### 3.2. Juvenile Dermatomyositis

JDM is a rare, autoimmune inflammatory myopathy of childhood that is characterized by proximal muscle weakness and distinctive cutaneous manifestations. The disease most commonly presents between 4 and 10 years of age and exhibits a marked female predominance, with a female-to-male ratio of approximately 2.5:1 [64,65].

#### 3.2.1. Clinical Manifestations

Proximal muscle weakness represents the key clinical manifestation of JDM and is frequently the first indicator of disease onset. It is typically symmetric, progressively worsens, and primarily involves the muscles of the shoulder and pelvic girdles. The clinical course is highly heterogeneous, ranging from mild muscle involvement to severe limitations in functional mobility [66].

**Figure 15:**
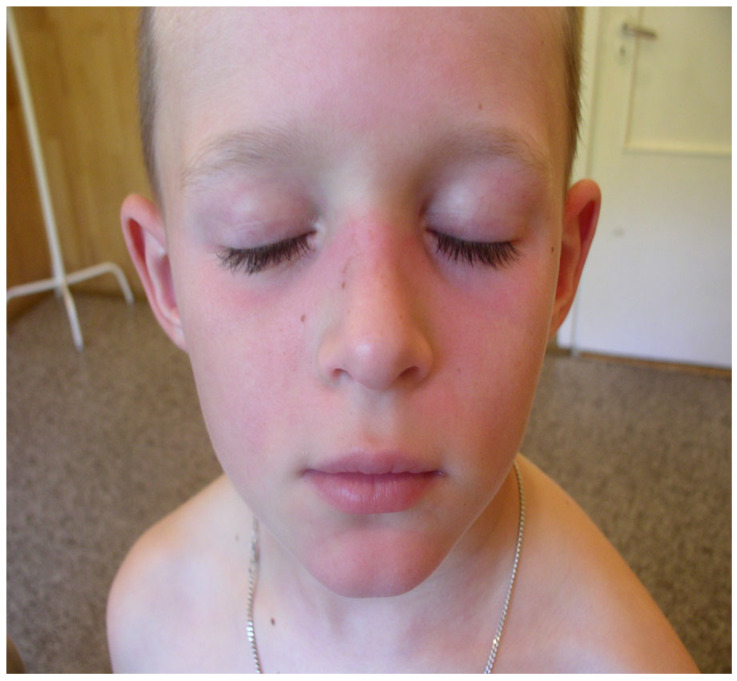
Cutaneous manifestations of JDM.

**Figure 16:**
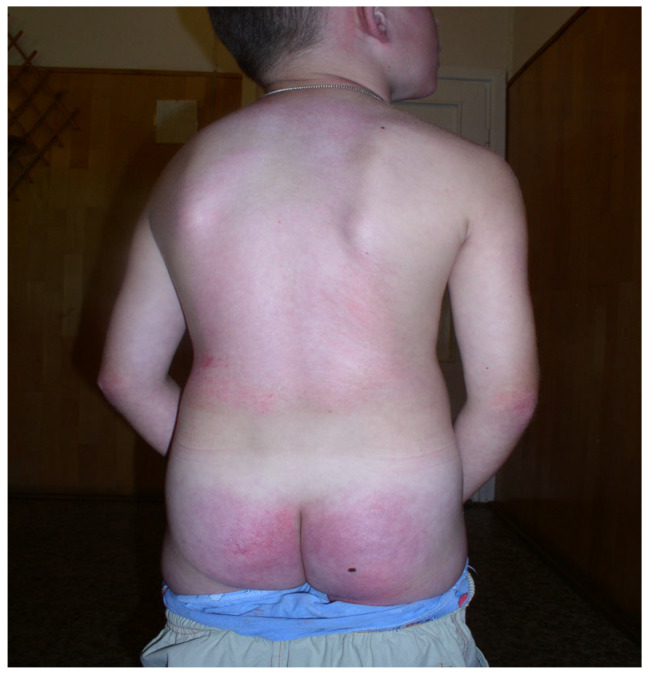
Cutaneous manifestations of JDM.

On physical examination, positive Gowers’ and Trendelenburg signs may be observed, and affected muscle groups may be tender to palpation or appear edematous. Involvement of palatal and pharyngeal muscles may result in voice disturbances, swallowing difficulties, hypernasal speech, and regurgitation [66]. Additionally, arthralgia and non-erosive inflammatory arthritis are commonly reported during the early phase of the disease [67]. Cutaneous involvement is a defining feature of the disease. Typical dermatological manifestations include heliotrope rash and Gottron’s papules [68]. Photosensitivity may present as erythema in sun-exposed areas, including the posterior neck and upper back (the “shawl sign”) as well as the anterior chest (the “V sign”) [66] (Figure 15 and Figure 16). 

In more severe cases, vasculopathy may lead to painful ulcerations, particularly involving the distal extremities. Nailfold capillary abnormalities indicative of small-vessel vasculopathy—such as capillary dilation and tortuosity—are commonly observed and have been shown to correlate with disease activity [69]. Gingival telangiectasia represents the most frequent oral manifestation and can be a valuable diagnostic indicator [70].

Calcinosis cutis occurs in approximately 34–70% of patients with JDM and is more prevalent in children than in adult-onset dermatomyositis [33,71]. Younger age at onset and prolonged disease activity are recognized risk factors for calcinosis [72]. Calcifications typically involve subcutaneous and muscular tissues, most commonly at sites exposed to pressure or trauma, such as the elbows, knees, and buttocks [73]. Lipoatrophy and scarring secondary to ulceration have been reported in approximately 30% of patients [74]. Systemic manifestations, including fever, fatigue, anorexia, malaise, and irritability, are frequently reported, especially during periods of increased disease activity [74]. The disease course is heterogeneous. Early disease onset has been linked to poorer outcomes in some reports, although evidence remains inconsistent. Unlike adult DM, JDM has not been clearly linked to malignancy [75].

#### 3.2.2. Autoantibodies

The autoantibody profile in JDM largely overlaps with that observed in adult dermatomyositis. Myositis-associated or myositis-specific autoantibodies are detected in approximately 70% of cases [76]. Anti-TIF1-γ antibodies are the most frequently identified (18–35%) and are associated with severe cutaneous involvement, including ulceration and lipodystrophy, as well as with greater muscle weakness. In contrast to adults, these antibodies are not associated with an increased risk of malignancy in pediatric patients [77,78,79]. Anti-MDA5 antibodies are detected in 6–38% of patients and are associated with arthritis, cutaneous ulcerations, and relatively mild muscle involvement. Importantly, this subgroup carries an increased risk of interstitial lung disease [78,79]. Anti-U1-ribonucleoprotein antibodies are more commonly observed in patients with later disease onset and are frequently associated with overlap phenotypes, particularly those exhibiting features of polymyositis and scleroderma-like characteristics [78,79]. Anti-Mi-2 antibodies, present in 4–10% of patients with JDM, are associated with clinical characteristics similar to those of adult patients. Despite severe histopathological findings, these patients generally respond well to standard therapy and have a favorable prognosis [23,78,79]. Anti-NXP2 antibodies (20–25%) are associated with severe muscle disease and an increased risk of gastrointestinal complications, including bleeding, ulceration, and dysphagia. The presence of this autoantibody significantly increases the risk of calcinosis in affected children. Patients with anti-NXP2 and anti-TIF1-γ antibodies are more likely to demonstrate resistance to conventional therapies [23,78,79].

#### 3.2.3. Histopathology

Skin biopsy in JDM most commonly demonstrates features of interface dermatitis and cutaneous microangiopathy. Frequent findings include vascular fibrin deposition (82%), perivascular lymphocytic infiltration (82%), and dermal edema (82%). In one study, extravasated erythrocytes, pigment incontinence, and thickening of superficial dermal vessel walls were observed in approximately 75% of specimens, while increased dermal mucin deposition and nuclear debris were present in 50%. Orthokeratosis was the predominant epidermal finding, followed by hyperkeratosis; parakeratosis was infrequently observed. Basement membrane thickening, epidermal atrophy, spongiosis, and superficial dermal fibrosis were identified in 25% of cases [80].

### 3.3. Amyopathic and Hypomyopathic Dermatomyositis

Amyopathic and hypomyopathic dermatomyositis are subtypes of idiopathic inflammatory myopathies and are characterized by the presence of typical cutaneous manifestations with absent or minimal involvement of skeletal muscle, and account for 20% of DM cases [81]. For many years, these entities were underrecognized or misclassified, despite their well-documented association with a significant risk of ILD and malignancy.

The term amyopathic dermatomyositis (ADM) is used when patients have typical dermatological signs of dermatomyositis, but without muscle involvement at all [82]. In patients with the cutaneous hallmarks of dermatomyositis and no clinically apparent muscle weakness, laboratory and electromyographic assessments may uncover subtle, subclinical muscle involvement. Such cases are designated as hypomyopathic dermatomyositis (HDM) or of CADM [82,83]. A study from the US showed that the incidence of CADM was 2.08 per 1 million persons. Patients with CADM were predominantly of adult-onset, female, and white race. Interestingly, muscle manifestations occurred at a mean of 6.85 years of follow-up in three out of nine patients [84].

#### 3.3.1. Clinical Manifestations

The first case reports of ADM come from Texas—six patients with classic cutaneous features of dermatomyositis who showed no clinical or laboratory evidence of muscle involvement for at least two years after onset of skin disease, accounting for 11% of all dermatomyositis cases. Inclusion criteria required pathognomonic skin findings (mostly Gottron’s papules), a biopsy consistent with dermatomyositis, and normal muscle enzymes and examination for two years. No malignancies were identified in this cohort [85]. ILD has been reported in patients with CADM, and many affected individuals develop rapidly progressive disease with poor outcomes.

In a Chinese retrospective cohort, ILD was present in 57% of CADM patients, most of whom developed rapidly progressive disease, with a 6-month survival of only 41% [86]. Kang et al. [87] reviewed 72 patients with PM/DM-associated ILD, including six with amyopathic DM. These ADM patients had typical DM rashes without muscle weakness or CK elevation. ILD developed in 29 patients, five of whom had ADM. Over a mean follow-up of 5.9 years, 11 patients died—mostly from ILD progression. CADM/ADM features were significantly associated with reduced survival among those with ILD [88].

#### 3.3.2. Histopathology

The hallmark histological features of dermatomyositis include vacuolar changes in the basal cell layer and mild to moderate mononuclear inflammatory infiltrates, a pattern referred to as interface dermatitis, along with increased dermal mucin. Epidermal atrophy may also be observed. Notably, the histological characteristics of amyopathic or hypomyopathic dermatomyositis are identical to those seen in classic dermatomyositis [89]. Compared to patients with HDM, ADM patients are less prone to epidermal atrophy and have a greater risk of mucin deposition [87].

#### 3.3.3. Antibodies

One study demonstrated that 65% (20 of 31 participants) with CADM tested positive for the anti-MDA5 antibody [52]. A subgroup of patients, who are positive with MDA-5, have a progressive course of the disease [90]. Recent studies have shown that initiating an early combined immunosuppressive regimen—comprising high-dose corticosteroids, intravenous cyclophosphamide, and calcineurin inhibitors—can improve survival in these patients [91]. Despite intensive standard therapy, 6-month mortality may reach as high as 50% [92]. Moreover, MDA-5-antibody-associated CADM is characterized by distinct cutaneous manifestations, such as mucocutaneous ulcerations, palmar papules, nonscarring alopecia, and panniculitis, which are thought to result from severe underlying vasculopathy [93]. As recently reported, anti-MDA5 dermatomyositis may present with refractory ulcerations in unusual anatomical sites, further broadening the recognized spectrum of cutaneous involvement [48]. Other antibodies that can also be found include SAE, TIF1-gamma, anti-Jo-1, anti-EJ, anti-PL-7, anti-PL-12, and anti-KS [94,95].

A functional MRI study demonstrated that patients with amyopathic dermatomyositis exhibited inefficient muscle metabolism during exercise compared with controls, whereas those with classic DM showed metabolic abnormalities even at rest [96].

In addition to the association with ILD, hypo/amyopathic DM patients appear to be at higher risk for internal malignancy. Udkoff et al. identified a subgroup of CADM patients in a Japanese cohort who were later diagnosed with malignancy. Among fifteen individuals with CADM, three had associated cancers—two lung cancers and one thymic tumor [97, 98, 99]. In amyopathic dermatomyositis, the malignancies most frequently reported are breast, lung, and ovarian cancers, whereas in classic dermatomyositis, the three most common cancers are lung, nasopharyngeal, and ovarian.

### 3.4. Paraneoplastic Dermatomyositis

Paraneoplastic syndromes are benign symptoms or conditions accompanying malignant neoplastic disease. They develop independently of local growth or the presence of distant metastases. In most cases, they are a consequence of a cross-immune reaction between neoplastic and healthy tissue, but they can also be caused by the tumor secreting biologically active substances such as hormones, peptides, or cytokines. The term cancer-associated myositis (CAM) refers to patients with DM who develop malignant tumors [4,7]. Studies have shown that the risk of developing malignant tumors in patients with DM is six times higher than in the overall population [4]. This increased risk, according to Scottish researchers, is highest in the first 3 months after DM diagnosis and lasts approximately five years from the time of diagnosis [7,98], but fortunately, it gradually decreases each year, although it never reaches the level observed in the general population [7,99,100]. According to various sources, between 15 and 42% of DM cases are associated with paraneoplastic syndromes, particularly in patients over 45 years of age, with the strength of this association increasing further after the age of 60 [100,101,102,103,104,105].

The most frequently diagnosed cancers coexisting with DM are breast cancer, ovarian cancer, lung cancer, and digestive system cancers (colon, rectum, and stomach cancer) [102,106,107]. However, this frequency may vary depending on the geographical region from which the patient originates. The best example of this phenomenon is that the malignancy most commonly associated with paraneoplastic dermatomyositis in Southeast Asia is nasopharyngeal carcinoma [101].

The guidelines of the International Myositis Assessment and Clinical Studies Group regarding cancer screening in patients with dermatomyositis recommend the use of several autoantibodies for risk stratification [108]. It is suggested that initial screening in all patients with newly diagnosed DM should include complete blood count, liver function tests, urinalysis, chest radiography, abdominal and pelvic ultrasonography, as well as age-appropriate investigations such as fecal occult blood testing, colonoscopy, gastroscopy, relevant tumor markers (at minimum, a PSA in men and CA-125 in women), mammography, transvaginal ultrasonography, and cytological examination [7,100,107,108]. Current scientific evidence recommends performing computer tomography only in cases where abnormalities are detected in the aforementioned investigations or in situations associated with an increased risk of malignancy [7,108]. It should be noted that studies aimed at the early detection of malignancies must be repeated in subsequent months; the most widely accepted schedule recommends that they should be performed no less frequently than once per year during the first three years.

Among all inflammatory myopathies, dermatomyositis exhibits the strongest association with malignancies. However, the pathomechanism underlying the relationship between dermatomyositis and cancer remains incompletely understood.

To date, no clinical, histological, or laboratory markers have been identified that are fully specific for the paraneoplastic form of dermatomyositis [102,109]. However, research has identified several autoantibodies that correlate with an increased likelihood of malignancy in patients with dermatomyositis. Among these are anti-TIF1γ antibodies, also referred to as p155/140 antibodies [4,7,108,110,111]. Their presence is significantly associated with an increased risk of malignancy, particularly in patients with dermatomyositis whose disease onset occurs after the age of 40 [112]. They demonstrate a sensitivity of 78% and a specificity of 89% in predicting subsequent cancer diagnosis, making them a valuable tool in screening investigations. Consequently, they may serve as potential prognostic markers indicative of adverse outcomes [113,114]. Recent studies suggest that anti-TIF1-γ-associated DM may arise as a consequence of an anti-tumor immune response, in which shared or cross-reactive antigens between tumor cells and regenerating muscle or skin tissue trigger autoimmune phenomena. This mechanism has been particularly highlighted in patients with hepatocellular carcinoma, where the development of DM has been linked to tumor-related immune activation [115].

Importantly, the presence of anti-TIF1-γ antibodies not only supports the need for thorough malignancy screening but may also carry prognostic implications. Emerging data indicate that the occurrence of TIF1-γ-associated DM in the context of cancer may be associated with a less favorable clinical outcome, further underscoring the importance of early recognition and interdisciplinary management. A positive correlation with malignancies, though to a lesser extent than for anti-TIF1, has also been observed in patients positive for NXP2 and SAE antibodies [4,7,40,108,110,111,116,117]. It has also been shown that certain serum autoantibodies may be associated with a reduced risk of malignancy. Results from several studies indicate that the presence of autoantibodies classically associated with myositis, such as anti-Jo-1, anti-Mi-2, and anti-U1-RNP, is linked to a low risk of cancer development in the course of dermatomyositis [109].

An increased risk of malignancy has been observed in male patients, in those of advanced age at the onset of dermatomyositis symptoms, and in patients presenting with dysphagia, vasculitis, V-shaped erythema, or an acute onset of cutaneous or muscular manifestations during the course of dermatomyositis [4,7,100,104,110,118,119]. There are also reports suggesting that the vesiculobullous variant of dermatomyositis is positively correlated with an increased risk of malignancy; however, due to the rarity of this form of DM, documenting and confirming this association remains challenging [120]. In contrast, the presence of interstitial lung disease, secondary Raynaud’s phenomenon, arthralgia, or arthritis, as well as a younger age at onset, has been associated with a lower risk of concomitant malignancy [119,121,122,123]. Unfortunately, the pathomechanism underlying these associations remains unexplained.

### 3.5. Overlap Myositis

Overlap myositis (OM), also referred to as myositis with overlap features, is now increasingly viewed as a distinct and diverse subtype of inflammatory myopathy. It is thought to comprise the largest proportion of the myositis group [124]. This term refers to patients who meet both criteria for DM/PM and other connective tissue diseases [125]. Reports indicate that between 11% and 40% of individuals with dermatomyositis also carry a concurrent diagnosis of a connective tissue disease [126]. Overlap disease tends to occur in younger patients and in females [125].

OM may occur alongside Sjögren’s syndrome, systemic sclerosis (SS), systemic lupus erythematosus (SLE), rheumatoid arthritis (RA), polyarteritis nodosa, and mixed connective tissue disease (MCTD) [126]. Among the OM subtypes, antisynthetase syndrome (ASS) is the most frequent and is often regarded as a distinct category within myositis. This syndrome is characterized by a recognizable constellation of features such as muscle inflammation, Raynaud’s phenomenon, arthritis, “mechanic’s hands,” ILD, and the presence of anti-tRNA synthetase autoantibodies [124].

Some resources showed that the most common associated disease with DM is scleroderma, and this variant is called sclerodermatomiositis. The most representative autoantibodies associated with the scleromyositis syndrome are the anti-PM/Sc and anti-survival of motor neuron (SMN) [127], which give a higher risk for calcinosis. Cutaneous manifestations can be heterogeneous, with patients presenting with oedematous or “puffy” hands, sclerodactyly with microstomia, or so-called mechanic’s hands. Ulcerations are relatively uncommon in this patient population [128].

In addition, patients frequently test positive for a range of non-myositis-specific antibodies, such as dsDNA, ANA, Scl-70, Jo-1, PM-Scl, Ku, and various extractable nuclear antigen antibodies. Laboratory evidence of skeletal muscle involvement includes increased levels of muscle enzymes such as creatine phosphokinase (CPK), aldolase, lactic dehydrogenase (LDH), and serum glutamic-oxaloacetic transaminase (GOT), with a frequency comparable to that observed in idiopathic DM [129].

In overlap syndrome, muscle inflammation is generally mild, although some patients may develop more pronounced symptoms. The most frequently observed manifestations include arthralgia or arthritis, sclerodactyly, Raynaud’s phenomenon, and myalgia, occurring in approximately 40–50% of patients [129]. Studies of hospitalized individuals with SLE suggested that confirmed inflammatory myopathy occurs in about 8% of cases. Importantly, myositis in overlap syndromes typically shows a stronger response to corticosteroids than in primary inflammatory myopathies [129].

Arthralgias, polyarthritis, and Raynaud’s phenomenon occur far more frequently in patients with overlap syndromes than in those with isolated dermatomyositis or polymyositis, or in individuals whose disease is associated with malignancy. The presence of a rash is higher in overlaps than in PM, but lower than in DM.

One study evaluated 12 ANCA-positive patients with polymyositis or dermatomyositis using the 2022 ACR/EULAR criteria for ANCA-associated vasculitis. Based on these criteria, 83.3% of patients met the definition of a PM/DM–AAV overlap syndrome, most commonly with microscopic polyangiitis. IDL, renal vasculitis, and ANCA positivity were the primary factors leading to classification [130].

### 3.6. DM Induced by Drugs

DM may be triggered or worsened by exposure to certain medications. DM that occurs in temporal association with exposure to a specific medication and cannot be attributed to other identifiable causes is defined as drug-induced dermatomyositis (DIDM) [131]. This condition may arise in patients with no previous history of autoimmune disease and can affect individuals of any age or sex [132].

Medications most frequently implicated in DIDM are hydroxyurea, followed by immune checkpoint inhibitors, statins, penicillamine, and tumor necrosis factor inhibitors [133].

Anne M. Seidler et al. [133] conducted a review of 70 cases from the literature to evaluate this topic. They showed that most of the cases were caused by hydroxyurea (51% of cases). All cases had pathognomonic signs, such as heliotrope rash, Gottron’s papules, or compatible cutaneous findings. Hydroxyurea cases lacked myositis, and the majority of cases had negative ANA. However, myositis was described in 79.4% of nonhydroxyurea cases. Of the patients, 44% had malignancy, mostly lymphoreticular cancers, and 16% had rheumatoid arthritis. The majority of cases of drug-induced DM improved after withdrawal of the medication, which was thought to play a role [131].

Statins can provoke a wide spectrum of myositis, including DM [134]. There are several cases in the literature that describe this correlation [135]. Clinical symptoms in these cases were classical: reddish rash on the face, Gottron’s papules, and heliotrope rash.

Biologic-induced DM is usually caused by PD-1 inhibitors, PD-L1 inhibitors, and CTLA-4 inhibitors, and most often develops within the first months of therapy [136]. All patients exhibited characteristic cutaneous manifestations, commonly the V-sign or heliotrope rash, while muscle weakness predominantly affected proximal limbs. ANA positivity was observed in 52% of cases, and elevated anti-TIF1-γ antibodies in 48%. Disease worsening after ICI rechallenge occurred in most cases, highlighting a likely causal role. Early recognition and timely treatment were crucial for optimal outcomes [137].

### 3.7. DM in Pregnancy

Reports describing the relationship between DM and pregnancy are rare, largely because the disease manifests during a woman’s reproductive years in only approximately 14% of cases [138].

Available data on the relationship between DM and pregnancy remain limited. Several mechanisms have been proposed as potential triggers for the development of DM during pregnancy [139]. Pregnancy-related alterations in humoral immunity, particularly a reduced antibody response to specific viral antigens, are potential mechanisms that link dermatomyositis with pregnancy.

Two types of pregnancy-related DM have been proposed: In the first type, disease activity is provoked during pregnancy and tends to improve after delivery, while in the other type, the onset is in the postpartum period, which is exceptionally rare [140]. The onset of dermatomyositis has been reported throughout pregnancy, with documented cases occurring in the first, second, and third trimesters [138,139,140].

Evidence from case reports and small observational studies suggests that women whose disease is in remission at the time of conception most often maintain disease stability throughout pregnancy and experience favorable obstetric outcomes [141,142,143].

In contrast, active DM present prior to conception or developing DM during pregnancy has been associated with increased risk of fetal death and preterm delivery, especially when complicated with active maternal myositis [144]. Morihara et al. reported that disease exacerbations in later stages of pregnancy (second and third trimesters) have been observed to correspond with better perinatal outcomes compared with flares in the first trimester [141].

### 3.8. Wong-Type Dermatomyositis

**Figure 17:**
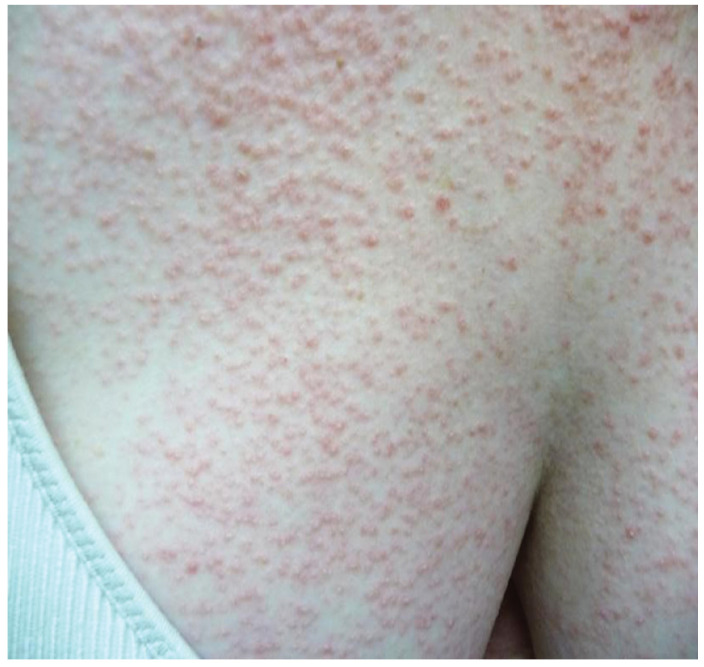
Wong-type dermatomyositis with hyperkeratotic follicular papules.

**Figure 18:**
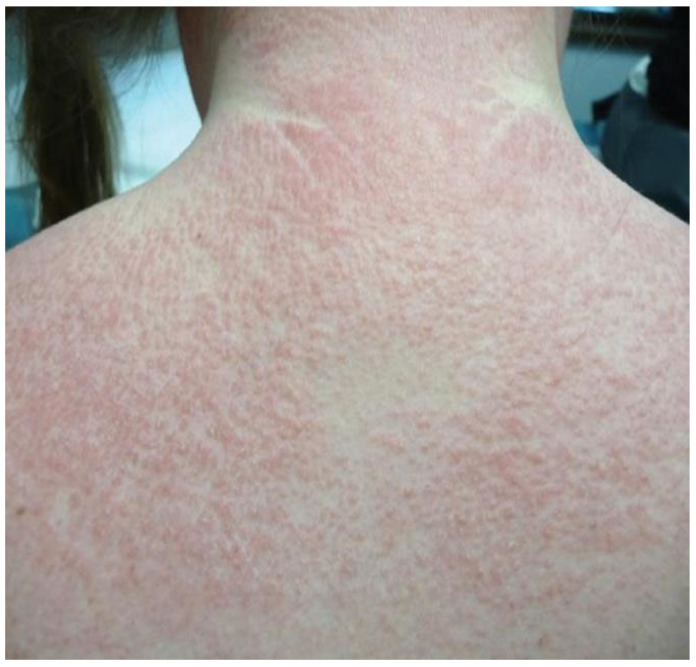
Wong-type dermatomyositis with hyperkeratotic follicular papules.

Wong-type of dermatomyositis (WTDM) is a rare clinical manifestation characterized by keratotic follicular papules that may mimic clinical symptoms of pityriasis rubra pilaris (PRP) [143]. The diagnosis of WTDM is usually delayed due to its rarity and atypical clinical presentation [145]. It can occur in both children and adults [146]. Wong’s type of dermatomyositis was named after the researcher Dr. K.O. Wong, who formally presented and discussed 11 clinical cases of WTDM in 1969 [147]. However, this rare clinical presentation of dermatomyositis was first described in 1953 by O’Leary, who reported a patient with erythroderma associated with dermatomyositis and plantar keratosis [148] (Figure 17 and Figure 18). 

To date, around 33 cases of WTDM have been described in the literature [30,149,150,151]. The clinical picture of Wong-type dermatomyositis is characterized by the presence of multiple, hyperkeratotic, erythematous, confluent follicular papules that tend to aggregate and form linear patterns on the bony prominences of the chest, back, upper and lower extremities [152,153,154]. Other manifestations of WTDM described include diffuse erythema or spinylosis, palmar-plantar keratosis, and porokeratosis-like lesions [155], typically characterized as atrophic patches or clusters of atrophic papules with a clearly marked, raised peripheral rim of scales. In addition to the characteristics of Wong-type DM, the patients also exhibited symptoms characteristic of the typical form of DM, such as heliotrope rash, Gottron’s papules, poikiloderma, and an erythematous rash in a shawl distribution [30,154,155,156]. Patients with WTDM usually also develop muscle symptoms. However, there were cases where these symptoms were not present [30,154,156]. Skin lesions in Wong-type DM may occur simultaneously, before, or after myositis [157]. Histological examination in WTDM reveals compact orthokeratosis, present in both follicular and non-follicular epidermal depressions. This often masks the subtle features that are characteristic of the classic form of DM, including vacuolization of the basal layer and mucin deposition [145]. Furthermore, arrector pilorum myositis is rarely present [143,157]. Another histopathological reaction pattern that is less frequently described in this subtype of DM is columnar dyskeratosis [158]. It is known that there is a certain correlation between the occurrence of DM and the risk of developing malignant tumors [159]. Still, knowledge about the relationship between WTDM and the presence of cancers is limited. Individual case reports describe the co-occurrence of Wong-type dermatomyositis with recurrent uterine cancer and fallopian tube cancer [154]. Cataldo-Cerda et al. described a case of a 51-year-old woman with a history of breast cancer who developed dermatomyositis showing symptoms of the Wong variant after completing adjuvant trastuzumab therapy [153]. The authors highlight that, as with the typical form of DM, this rare subgroup should be considered a potential paraneoplastic dermatosis [154]. When a Wong-type of dermatomyositis is suspected, the overlapping symptoms of DM and pityriasis rubra pilaris (PRP) should be considered in the differential diagnosis [146].

### 3.9. Antisynthetase Syndrome

Antisynthetase syndrome (ASS) is currently regarded as a distinct disease entity within the spectrum of IIM. However, due to substantial overlap in clinical and immunological features, it is frequently discussed in the context of DM or PM and, according to certain classification criteria (e.g., the EULAR/ACR criteria for inflammatory myopathies), may even be classified as DM or PM [5,160,161]. Detailed knowledge of this disease enables its differentiation from other conditions associated with muscle involvement, as well as from overlap syndromes and, consequently, helps to avoid misdiagnosis.

ASS is a rare, chronic, systemic disease in which the classic clinical presentation comprises three features: myositis, arthritis, and interstitial ILD [161,162]. A defining characteristic of the syndrome is the presence of autoantibodies directed against aminoacyl-tRNA. Additional clinical manifestations typically include so-called “mechanic’s hands,” Raynaud’s phenomenon, and fever; however, the clinical spectrum is broad.

In the 1980s, autoantibodies directed against aminoacyl-tRNA synthetases were described and linked to idiopathic inflammatory myopathies [18]. Subsequently, Witt et al. demonstrated an association between anti-Jo-1 antibodies and interstitial lung disease, which allowed ASS to be distinguished as a separate clinical entity [163]; however, it was not until 2010 that Wells et al. proposed formal diagnostic criteria for antisynthetase syndrome [164]. Although ASS was first described approximately 40 years ago, its prevalence remains incompletely understood, and its pathogenetic mechanisms remain unclear [163,165].

The global prevalence of ASS is estimated at approximately 1–9 per 100,000 individuals; however, available epidemiological data remain limited [166]. ASS occurs more frequently in women; the estimated female-to-male ratio is approximately 7:3, and the mean age at disease onset, reported to be 48–51 years, is comparable to that observed in patients with dermatomyositis [167]. Available data indicate that ASS is exceptionally rare in the pediatric population [168].

The EULAR/ACR developed classification criteria for idiopathic inflammatory myopathies and their major subgroups; however, ASS was not distinguished as a separate entity. Consequently, two principal sets of criteria for ASS—the Solomon criteria and the Connors criteria—have been proposed, both of which require the presence of antisynthetase antibodies (ASAb) as a mandatory condition for diagnosis [6,164]. The remaining diagnostic criteria for ASS according to Connors are presented in Table 2. The fact that the EULAR/ACR criteria do not take into account antibodies other than anti-Jo-1 may lead to underestimation of ASS diagnoses [6]. Moreover, due to the considerable heterogeneity of the clinical presentation of ASS and the observation that not all characteristic features are present in every patient, establishing an accurate diagnosis can be challenging, even when applying the Solomon or Connors criteria. This ultimately leads to delays in diagnosis and treatment. For example, among patients with anti-Jo-1 antibodies, the mean time to diagnosis was approximately six months [169], whereas individuals positive for anti-PL-7 or anti-PL-12 antibodies experienced an even longer diagnostic delay [165].

Nailfold capillaroscopy is a simple, noninvasive method that allows assessment of microangiopathic changes and differentiation between primary and secondary Raynaud’s phenomenon. In the analysis by Legout et al., abnormal capillaroscopic findings were observed in over 60% of patients with ASS, including giant capillaries, microhemorrhages, branching, and avascular areas [170]. The latter, seen in nearly 18% of patients, was associated with myositis, the presence of anti-SSA and anti-Jo-1 antibodies, and Raynaud’s phenomenon. A scleroderma-like pattern was described in more than one-third of patients, more frequently in those with longer disease duration or anti-Jo-1 positivity; interestingly, it was also observed in about one-third of ASS patients without Raynaud’s phenomenon, highlighting the utility of capillaroscopy as a supportive diagnostic tool in ambiguous cases.

Anti-Jo-1 antibodies are typically associated with the classic triad of ASS features, but the anti-Jo-1-positive group more frequently exhibited the “mechanic’s hands” sign and lower serum CK levels [95]. Another analysis demonstrated that in patients with anti-Jo-1 antibodies, Raynaud’s phenomenon was the initial manifestation in as many as 17% of cases [169]. Japanese researchers demonstrated that fewer than 10% of patients with positive anti-Jo-1 ASS exhibited heliotrope rash, compared with approximately 20–30% of those with anti-EJ, anti-PL-7, or anti-PL-12 antibodies. In contrast, the frequency of Gottron’s sign was similar across anti-Jo-1 and other anti-ASAb-positive subgroups [95]. The presence of these antibodies is generally associated with an earlier diagnosis of ASS and improved overall survival, likely due to a prompt initiation of treatment [171,172].

Anti-PL-7 and anti-PL-12 antibodies in ASS exhibit a similar phenotype and are therefore often discussed and studied together. Available analyses have reported inconsistent results regarding the association between Raynaud’s phenomenon and serological profile. In a study published in 2019, Raynaud’s phenomenon occurred most frequently in the anti-PL-12-positive subgroup compared with other ASAb-positive subgroups [173]. On the other hand, Aggarwal et al. suggested a higher prevalence of this manifestation in patients with anti-PL-7 antibodies [174]. In another study, no statistically significant differences were observed in the occurrence of Raynaud’s phenomenon between patients positive for anti-Jo-1 antibodies and those positive for anti-PL-7 or anti-PL-12 antibodies. The same analysis also revealed no differences in the frequency of “mechanic’s hands” or fever [175]. Unfortunately, patients presenting with the anti-PL-7/PL-12 ASS phenotype received a diagnosis later than those positive for anti-Jo-1, which was often associated with shorter survival [173].

Anti-OJ antibodies are among the rarest identified antisynthetase antibodies, most likely due to the need for immunoprecipitation rather than other, more readily available laboratory methods [174,175]. Consequently, data on cutaneous manifestations in patients with ASS and positive anti-OJ are limited. A review of 52 published cases of ASS with anti-OJ antibodies reported a prevalence of interstitial lung disease of 90%, myositis of 75%, mechanic’s hands of 36%, and arthritis of 31% [176].

ASS with anti-EJ antibodies typically initially manifests as isolated ILD. Analysis of 51 patients with anti-EJ associated ASS-ILD showed that the most common extrapulmonary symptom is mechanic’s hands, observed in 43.1% patients, while Gottron’s sign nwas observed in 25.5%, heliotrope symptoms in 13.7%, and Raynaud’s phenomenon in 3.9% of patients. It follows that mechanic’s hands and Gottron’s sign in those patients are observed more often than in ASS-ILD anti-EJ-negative type, while heliotrope symptoms and Raynaud’s phenomenon are found less frequently [177]. Vulsteke et al. reported similar findings, with Gottron sign observed in 31% and heliotrope rash in 16% of patients with anti-ARS antibodies, but these manifestations were most frequent in patients with anti-EJ, anti-PL-7, and anti-PL-12 antibodies, with heliotrope rash occurring in approximately 20–30% of anti-EJ-positive cases [178].

Data on anti-KS, anti-Ha, and anti-Zo antibodies in ASS are also limited, primarily to case reports and small case series, as detection of these autoantibodies—similar to anti-OJ—requires immunoprecipitation, which is not widely available. Overall, patients with these antibodies in the course of ASS present a clinical picture similar to other ASS subtypes [178]. Anti-Ro antibodies, including anti-Ro52, are the most frequently occurring (30–65%) coexisting antibodies in patients positive for ASAb, and although they are not antisynthetase antibodies, they may influence the course of ASS [178,179]. Although Ro-52 antibodies are typically regarded as myositis-associated, patients with isolated Ro-52 positivity have also been reported to exhibit clinical features such as mechanic’s hands and ILD [175].

ASS primarily manifests with the classic triad of ILD, arthritis, and myositis, although patients often present with only one feature initially. In anti-Jo-1-positive patients, about half develop the remaining triad elements over months to even years [169]. While some manifestations are more frequent with specific ARS antibodies, the overall phenotype is largely similar. Euro-Myositis Registry data indicate muscle involvement (90%) and ILD (71%) as the most common features, though prevalence may vary with cohort characteristics and rare antibody subgroups [167].

Beyond the classic triad of symptoms, the clinical picture of ASS may include a range of other manifestations, such as pulmonary hypertension, fever, dysphagia, regurgitation, myocarditis, pericarditis, as well as less or more characteristic cutaneous features.

A characteristic cutaneous manifestation of ASS is the so-called mechanic’s hands. This term refers to erythematous, cracked, and hyperkeratotic skin changes resembling dermatoses seen in individuals performing manual labor, without associated pruritus. They typically localize on the lateral aspects of the hands and fingers as well as the fingertips, usually sparing the dorsal and palmar surfaces of the fingers [97]. These changes are dynamic in nature—they improve with systemic treatment and worsen during periods of increased disease activity [19,179]. Their prevalence among patients with ASS is estimated at 16–21%. In various studies, the prevalence of this feature ranged from 19% to 56.5% of patients with ASAb. Although mechanic’s hands are most commonly associated with ASS, they are not specific and may also occur in other forms of inflammatory myopathies or mimic other dermatoses [180].

Some reports indicate that mechanic’s hands are associated with a higher prevalence of ILD in ASS [181] and are more often in patients with anty-Jo-1 ASS [182]. Different results were carried out by Bachmeyer et al., who found that patients positive for anti-PL12 antibodies most frequently exhibited mechanic’s hands among ASAb-positive patients [183]. In recent years, similar lesions localized on the feet, referred to as “hiker’s feet”, have been described, which are morphologically analogous to mechanic’s hands [184,185]. These lesions were observed in the majority (89%) of patients who exhibited mechanic’s hands [186].

Unlike DM, ASS is not clearly associated with increased malignancy risk. Small reports suggest incidences up to 14% [187,188,189], but larger cohorts report rates similar to the general population (1.7%) [190].

Considering both the typical and less characteristic manifestations of ASS, it should be noted that approximately 5–8% of ASS cases present as overlap syndromes with other connective tissue diseases, such as systemic lupus erythematosus, systemic sclerosis, or Sjögren’s syndrome [172].

### 3.10. Flagellate Dermatitis

Flagellate dermatitis is an uncommon skin disorder characterized by nonspecific inflammatory features, including linear or curvilinear streaks and plaques, which are similar to the marks of whiplashes. Those skin lesions predominantly affect the trunk and are typically accompanied by intense itchiness. It is most commonly caused by exposure to bleomycin or by consuming raw or undercooked shiitake mushrooms [191,192].

**Figure 19:**
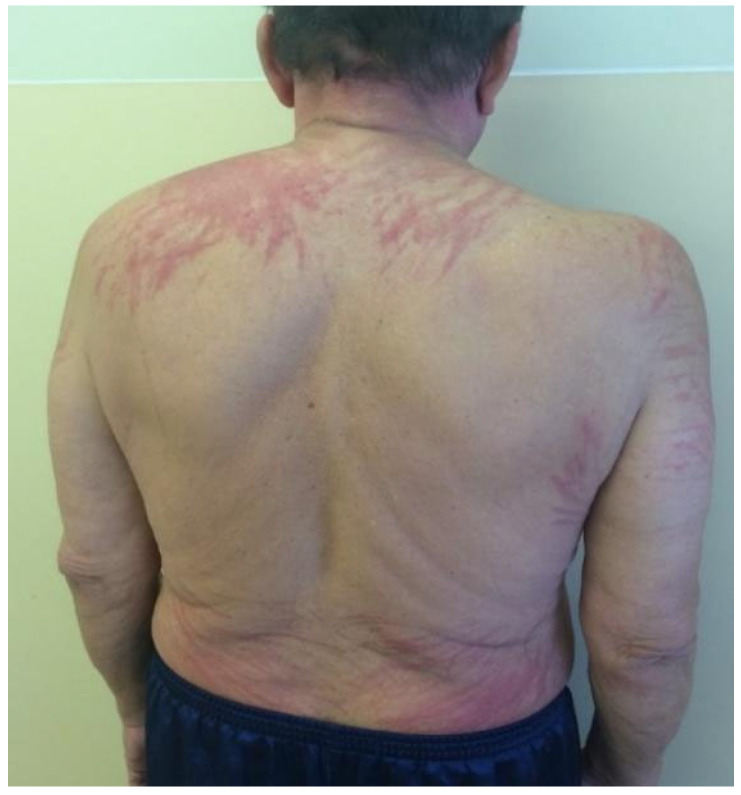
Patchy and linear erythematous-edematous lesions on upper back.

**Figure 20:**
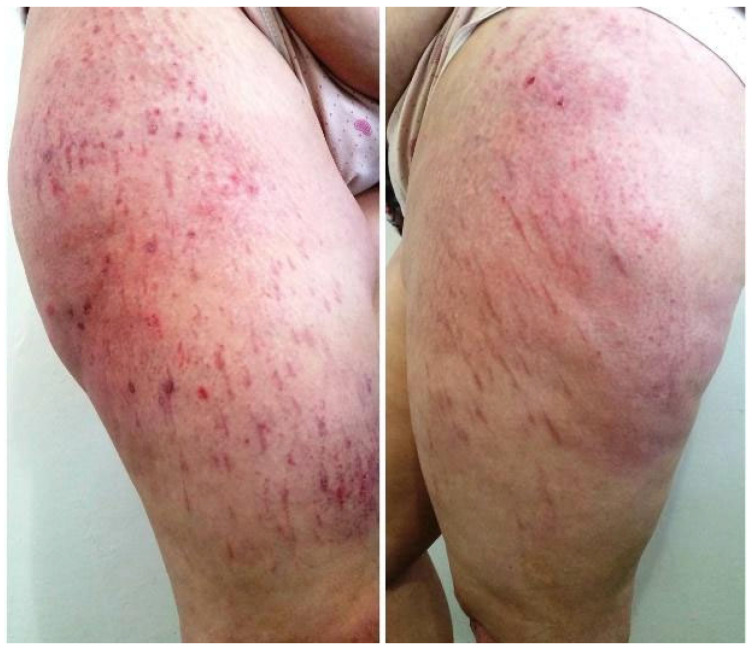
Patchy and linear transverse erythematous-edematous lesions on thighs.

Flagellate dermatitis is an extremely rare clinical form of dermatomyositis. A few cases of characteristic clinical symptoms of flagellate dermatitis in the course of DM have been reported. The first description of linear changes in patients with dermatomyositis was presented in a paper by Bohan et al. in 1975 [3]. Patients with dermatitis in the course of dermatomyositis present with patchy and linear transverse erythematous-edematous lesions in the back area. These lesions may be accompanied by intense pruritus [193,194,195] (Figure 19 and Figure 20). In addition, patients often also experience characteristic skin symptoms such as heliotrope rash, Gottron’s papules, shawl sign, the V sign, periorbital oedema, periungual telangiectasia, and mild poikiloderma of the upper back. Muscular symptoms such as weakness and pain may also occur [193,194,196].

Watanabe et al. reported a case of a patient with amyopathic dermatomyositis who had typical DM lesions such as heliotrope rash, Gottron papules, and Gottron sign, which co-occurred with flagellate dermatitis-type lesions [197]. These changes presented as a long flagellate streak and large, erythematous lesions in the form of red papules that were arranged in a linear pattern across the lower back and resembled whiplash marks. The pathogenesis of flagellate dermatitis remains unclear, but possible contributing factors include mechanical trauma such as abrasions and minor skin damage, and exposure to sunlight [194]. It has been suggested that this subtype of DM may indicate the presence of malignant tumors in internal organs [193].

### 3.11. Degos-like Dermatomyositis

Degos disease is a rare microangiopathic disorder of unknown pathogenesis. There are two subtypes of this disease: malignant atrophic papulosis (MAP), which usually leads to death, and a skin-limited form, benign atrophic papulosis (BAP). The clinical presentation of the disease initially includes small, erythematous papules, which then undergo central collapse, forming characteristic porcelain-white atrophic foci that are surrounded by an erythematous, telangiectatic rim. Lesions are mainly located on the trunk and upper limbs, plantar, scalp, and face [198,199]. Characteristic findings in histopathological examination of the tissue include hyperkeratosis, epidermal atrophy, separation of the dermis and epidermis, and wedge-shaped fibrotic necrosis of tissues caused by thrombotic occlusion of small arteries deep in the dermis [199,200]. Pathological examination revealed that the microscopic features of Degos disease and dermatomyositis show similar deposits and vascular patterns [201]. To date, individual cases of Degos dermatomyositis have been reported. Clinical symptoms in patients include changes characteristic of typical DM, such as muscle pain, heliotrope rash, and Gottron’s papules, shawl-like erythema, as well as changes typical of Degos disease associated with microcirculation disorders in the skin, gastrointestinal tract, and other organs [201]. Skin lesions in the course of DM of the Degos type usually present as concave, porcelain-white papules or atrophic patches surrounded by a thin rim of erythema or on a reticular background [202]. Skin lesions are accompanied by pain or itching and appear on the abdomen, chest, back, face, and upper and lower limbs. Changes typical of Degos disease usually appear for the first time several months after the diagnosis of dermatomyositis [203].

### 3.12. New Biomarkers in Dermatomyositis

Research is constantly being conducted on the development of new biomarkers that will be used to diagnose and predict the course of DM and associated diseases. As accurate analysis of disease activity is crucial for assessing the course of the disease, new and increasingly sensitive and specific biomarkers are constantly being sought to enable more precise monitoring. One such biomarker is sialic acid binding Ig-like lectin 1 (SIGLEC1), which has proven useful in monitoring disease activity and response to treatment in both juvenile and adult DM [204,205]. Serum interferon type I (IFN-I) levels have also been described as a potential indicator of skin disease activity in patients with juvenile dermatomyositis [205]. Furthermore, another study has demonstrated the reliability of galectin-9 and CXCL10 as markers for assessing disease activity, highlighting their significant potential for clinical application in everyday practice. Molecules such as LY6E, IFITM1, GADD45A, MT1M, and SPP1 have been identified as potential predictive biomarkers of newly diagnosed hepatocellular carcinoma (HCC) in patients with dermatomyositis [206,207,208].

## 4. Conclusions

Dermatomyositis is a heterogeneous disease with a broad and complex spectrum of clinical manifestations and remains associated with several poorly understood aspects, particularly with regard to its etiopathogenesis, diagnosis, and treatment. As a systemic condition affecting multiple organs, it requires awareness among physicians from various medical specialties of its diverse morphological presentations. The fact that cutaneous lesions may constitute the sole clinical manifestation of the disease underscores the critical role of thorough dermatological assessment in the diagnostic process of dermatomyositis. Accurate evaluation of the full spectrum of pathological manifestations, including skin lesions ranging from pathognomonic and characteristic to rare and nonspecific findings, is essential for proper differentiation from other disease entities, thereby enabling early diagnosis and timely initiation of appropriate therapy. Advances in the understanding of the immunological mechanisms underlying dermatomyositis have allowed the identification of associations between specific clinical subtypes and distinct serological markers. Consequently, the determination of a targeted autoantibody profile—often linked to particular clinical phenotypes—may aid in prognostic assessment and therapeutic decision-making. Given the complexity of the disease, an interdisciplinary approach and close collaboration among specialists from multiple medical disciplines are essential to ensure optimal and comprehensive patient care.

## Figures and Tables

**Table 1:** European League Against Rheumatism (EULAR) with the ACR classification criteria for IIM.

Variable	Score in Case of Muscle Biopsy	Score with No Muscle Biopsy
Onset between the ages of 18 and 40 years	1.5	1.3
Onset over the age of 40 years	2.2	2.1
**Heliotrope syndrome**	3.2	3.1
**Gottron’s papules**	2.7	2.1
**Gottron’s symptom**	3.7	3.3
More pronounced weakness of neck flexors than extensors	1.6	1.9
Objective symmetrical reduction of strength of proximal muscles in upper extremities	0.7	0.7
Objective symmetrical reduction of strength of proximal muscles in lower extremities	0.5	0.8
Proximal muscles in lower extremities weaker than distal ones	1.2	0.9
Dysphagia or disorders of peristalsis of the esophagus	0.6	0.7
Anti-Jo-1 antibodies	3.8	3.9
Increased activity of creatine phosphate kinase (CPK) or lactic acid dehydrogenase (LDH), or serum alanine aminotransferase (ALT) or aspartate aminotransferase (AST)	1.4	1.3
Inflammatory infiltration composed of mononuclear cells within the endomysium	1.7	-
Perivascular and perimuscular infiltrations composed of mononuclear cells	1.2	-
Peripheral vacuolization of muscle cells	3.1	-
Peripheral atrophy of muscle fibers	1.9	-

**Table 2:** Connor et al.’s diagnostic criteria for antisynthetase syndrome (2010) [166].

Required	Presence of an Anti-Aminoacyl-tRNA Synthetase Antibody
Plus one or more of the following clinical features	Raynaud’s phenomenon Arthritis Interstitial lung disease Fever (not attributable to other causes) Mechanic’s hands

## Data Availability

The original contributions presented in this study are included in the article. Further inquiries can be directed to the corresponding author.

## Abbreviations

ACR	American College of Rheumatology
ADM	Amyopathic Dermatomyositis
ANA	Antinuclear Antibodies
ANCA	Antineutrophil Cytoplasmic Antibodies
ASAb	Anti-tRNA Synthetase Antibodies
ALT	Alanine Aminotransferase
AST	Aspartate Aminotransferase
CADM	Clinically Amyopathic Dermatomyositis
CAM	Cancer-Associated Myositis
CK/CPK	Creatine Kinase/Creatine Phosphokinase
CTLA-4	Cytotoxic T-Lymphocyte-Associated Protein 4
DIDM	Drug-Induced Dermatomyositis
DM	Dermatomyositis
EULAR	European League Against Rheumatism
EMG	Electromyography
HDM	Hypomyopathic Dermatomyositis
HRCT	High-Resolution Computed Tomography
IBM	Inclusion Body Myositis
ICI	Immune Checkpoint Inhibitors
IIM	Idiopathic Inflammatory Myopathies
ILD	Interstitial Lung Disease
JDM	Juvenile Dermatomyositis
LDH	Lactate Dehydrogenase
MAA	Myositis-Associated Autoantibodies
MCTD	Mixed Connective Tissue Disease
MRI	Magnetic Resonance Imaging
MSA	Myositis-Specific Autoantibodies
NXP-2	Nuclear Matrix Protein 2
OM	Overlap Myositis
PD-1	Programmed Death-1
PD-L1	Programmed Death Ligand-1
PM	Polymyositis
PDM	Paraneoplastic Dermatomyositis
RA	Rheumatoid Arthritis
SAE	Small Ubiquitin-like Modifier Activating Enzyme
SLE	Systemic Lupus Erythematosus
TIF1-γ	Transcription Intermediary Factor 1-gamma
